# Rheological properties of calcite oozes: Implications for the fossilisation in the plattenkalks of the Solnhofen-Eichstätt lagoons in the Franconian Alb, Germany

**DOI:** 10.1371/journal.pone.0252469

**Published:** 2021-06-02

**Authors:** Sydney Gerschermann, Chris Ballhaus, Fabian Gäb

**Affiliations:** 1 Institut für Geowissenschaften, Universität Bonn, Bonn, Germany; 2 Geographisches Institut, Universität zu Köln, Köln, Germany; Southern Illinois University, UNITED STATES

## Abstract

We report on an experimental study to investigate the sedimentation behaviour and rheological properties of extremely fine-grained calcite oozes. The experiments are aimed at clarifying if thixotropic behaviour may have played a role in the preservation of marine biota in plattenkalks of the Solnhofen lagoons of the Franconian Alb. Calcite particles with grain sizes from 2.2 to 4.4 μm were sedimented from water, seawater proxies, and hypersaline brines with up to 14 wt.% NaCl, for 170 days. High salinities as envisioned for the bottom waters of some Solnhofen lagoons slow down settling rates of calcite and may produce plattenkalks more porous and more friable than plattenkalks elsewhere in the Solnhofen archipelago. Rheological properties of calcite suspensions were measured with an oscillation rheometer. Calcite oozes with 40 vol.% calcite in suspension behave thixotropically regardless of the salinity of the pore solutions. Thixotropic behaviour may have the potential to promote the fossilisation of marine biota. Even if the sediment cover is thin, i.e. a few millimeters, a carcass covered by a thixotropic sediment would be largely isolated from the overlying water column because pore solutions in thixotropic media hardly communicate with the overlying water column. A fish carcass covered by a thixotropic sediment could impose local-scale physicochemical conditions on its direct sedimentary envelope favourable for preservation and the replacement of organic material by inorganic materials.

## Introduction

**Fig 1:**
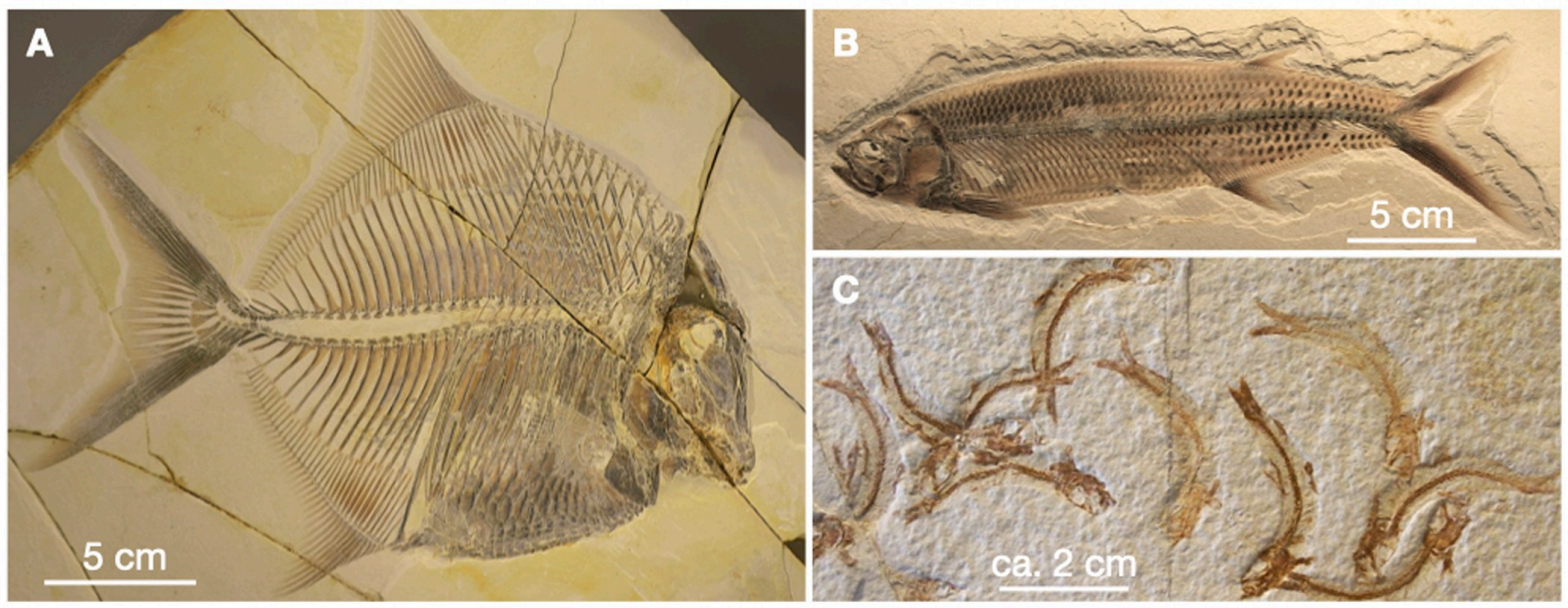
Fish fossils from the Solnhofen lagoons to illustrate the excellent preservation. (A)—an Upper Tithonian Pycnodontid (*Proscinetes elegans*) from the Ettling quarry (JME-ETT876), Frankonian Alb [6]. (B)—*Thrissops cf*. *formosus* from Ettling (JME-ETT74) with colour pattern preservation [5]. (C)—Species of *Leptolepides sprattiformis* [7] from Solnhofen (JME-SOS8059). All photos from the second author, with permission from the Jura-Museum Eichstätt.

The lithographic limestones of the Solnhofen-Eichstätt area in the Franconian Alb, subsequently referred to as Solnhofen, carry the best-preserved fossils worldwide. The plattenkalks of the Solnhofen area are the type locality for 12 specimens of *Archaeopteryx*, for many pterosaurs [1], diverse invertebrates, and a plethora of fish [2]. Overall, fossils are rare, but preservation is outstanding. Many fish exhibit soft tissue preservation and are fully articulated [3], and some even preserve pigmentations that are interpreted as original colour patterns [4, 5]. The best preservations show fossils that were embedded in micritic plattenkalks (Fig 1).

A common notion is that successful fossilisation requires that a carcass be covered rapidly by sediment [8–10]; that it be isolated quickly from the water column above before decomposition (oxidised) or putrefaction (anoxic) set in. Rapid isolation can happen by turbidity currents, examples being the fossils of the Hunsrück-Schiefer and Beecher’s trilobite bed [11–14]. Rapid sedimentation can imprint anoxic to euxinic conditions on the sediment around a carcass even if the overlying seawater is oxidised, hence can promote the replacement of organic matter by inorganic materials [15]. However, not everywhere where high-quality fossils are preserved, average sedimentation rates were high. For the plattenkalks of Solnhofen, Barthel [16] and Park and Fürsich [17] envisioned average sedimentation rates of around 0.1 to 0.5 mm per year. At such low rates we may have to search for parameters that allow a fish carcass to remain on the sediment-water interface without decay, at least until it is covered by sediment.

To address that issue, Gäb et al. [18] carried out taphonomic experiments to simulate conditions for good conservation before a fish was embedded in sediment. They show that the most effective parameters are [1] elevated hydrostatic pressure or water depth to allow a fish after its death to sink in the water column and settle on the seafloor; [2] elevated salinities around 3 times seawater and/or elevated pH > 9 to inhibit bacterially mediated decay; [3] rapid encapsulation by bacterial mats [19], and [4] anoxic conditions. Anoxia *per se* does not stop the decay of organic matter [18] but it does create an environment in the bottom waters hostile toward scavengers.

**Fig 2:**
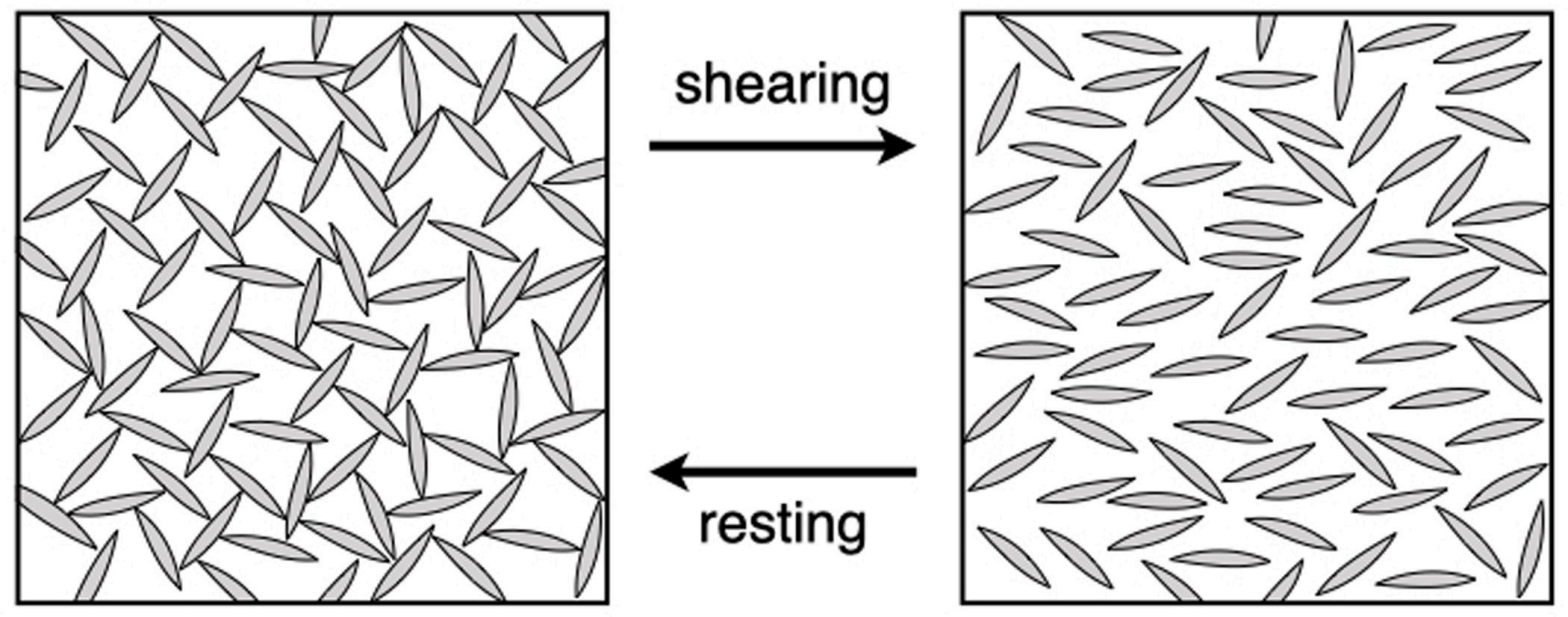
Breakdown of a thixotropic structure with shear stress. Modified from Barnes [27].

For later fossilisation, however, sedimentation is important. Some sedimentary features of the Solnhofen plattenkalks also show that an increased sedimentation must have taken place at times. Taking this into account, Keupp et al. [20] and Viohl [21, 22] assume a model containing a short-term sedimentation in which summer storm events lead to the mobilization of calcite and thus to a high and rapid sedimentation. But what mechanisms are decisive here? What role did the physical properties of the sediments play in Solnhofen? The plattenkalks are extremely fine-grained and their precursor sediments—micritic calcite suspensions—could have been thixotropic because the rheology of substances is decisively influenced by grain size [23–25]. The proposition that calcite oozes may be thixotropic is not new [26], but has not been followed up in the subsequent Solnhofen literature as a taphonomic factor. Similar to bacterial mats [19], thixotropic sediments can effectively isolate a carcass from the overlying water column, even if the sediment cover is still thin, with thicknesses on the millimeter scale. Thixotropic substances behave like solids when they are at rest, and the circulation of pore solutions inside thixotropic media and their exchange with the overlying water column is strongly inhibited by a cross-linking structure between grains and pore solution via hydrogen bonds (Fig 2). A fish carcass embedded in a thixotropic medium could impose its own small-scale physicochemical conditions on its surrounding sediments through local decay of organic matter, thus promoting its own conservation.

In this paper we investigate the sedimentation behaviour and the rheological properties of micritic calcite oozes, as they may have occurred in the Upper Jurassic of Solnhofen when the plattenkalks were deposited. Calcite oozes with grain sizes between 2.2 and 4.4 μm were sedimented in volumetric flasks, and porosities were monitored over time. To investigate possible thixotropic behaviour, the rheological properties of three calcite suspensions were investigated with an oscillation rheometer. It has long been suspected that the bottom waters of some Solnhofen lagoons were hypersaline [21], and therefore salinities of the experimental calcite suspensions were varied between zero and 14 wt.% NaCl. The results indicate that the settling rates of calcite from suspensions depend on the salinity of the solutions. All CaCO_3_ oozes whose rheological properties were investigated show thixotropic behaviour. We suggest that in addition to salinity, pressure, and overgrowth by bacterial mats [18], the rheological properties of the sediments may have played an important role for the excellent preservation of fish in the Solnhofen lagoons.

### The Solnhofen archipelago in the Upper Jurassic

**Fig 3:**
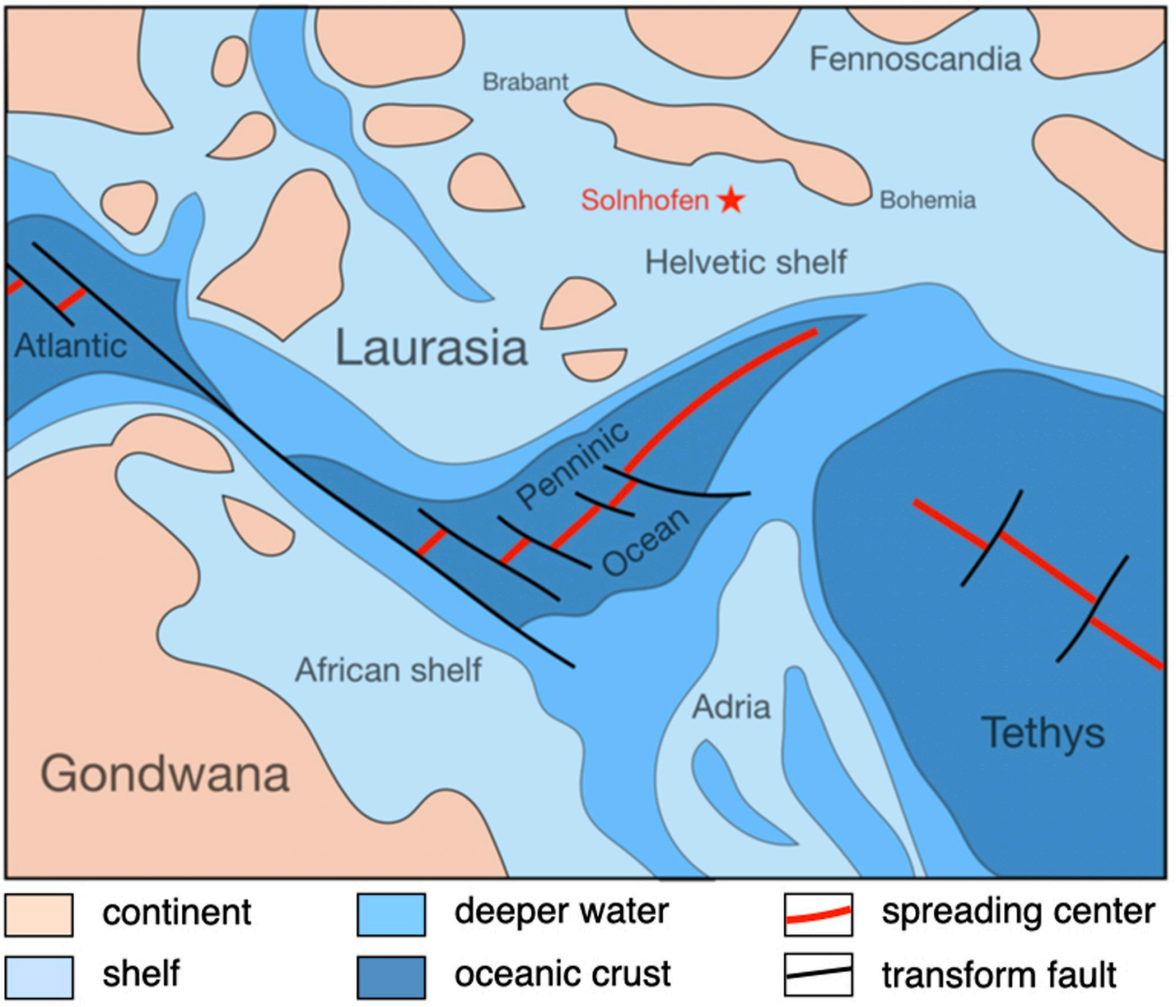
Palaeogeography around the Helvetic shelf in the Upper Jurassic. Modified from Schuster et al. [30].

The sediments at Solnhofen with the best-preserved fossils are well bedded plattenkalks with calcite grain sizes < 4 μm. They were deposited around 150 Ma ago in km-sized lagoons situated on the Helvetic shelf. On a regional scale, the Franconian Alb in the Upper Jurassic was a shelf area at about 30° N, bordered to the south by the Penninic ocean and to the north by the London-Brabant, the Rhenish, and the Bohemian massifs (Fig 3). On the shelf, sponge and coral reefs alternated laterally on a km scale with deeper basins or lagoons in which micritic calcite sediments were deposited. Some lagoons may have been temporarily isolated from the open sea and chemically stratified, with anoxic hypersaline bottom brines overlain by oxidised normal-salinity seawater [21, 28]. The climate in the Upper Jurassic was more balanced than today. Hallam [29] assumed that the distribution of continents and seas around Solnhofen—Fennoscandia and Laurasia to the north, Gondwana to the southwest, and the Penninic ocean and Tethys to the southeast (Fig 3)—caused a monsoon-type climate with moist southeasterly onshore winds in summer and dryer offshore westerly winds in winter times.

**Fig 4:**
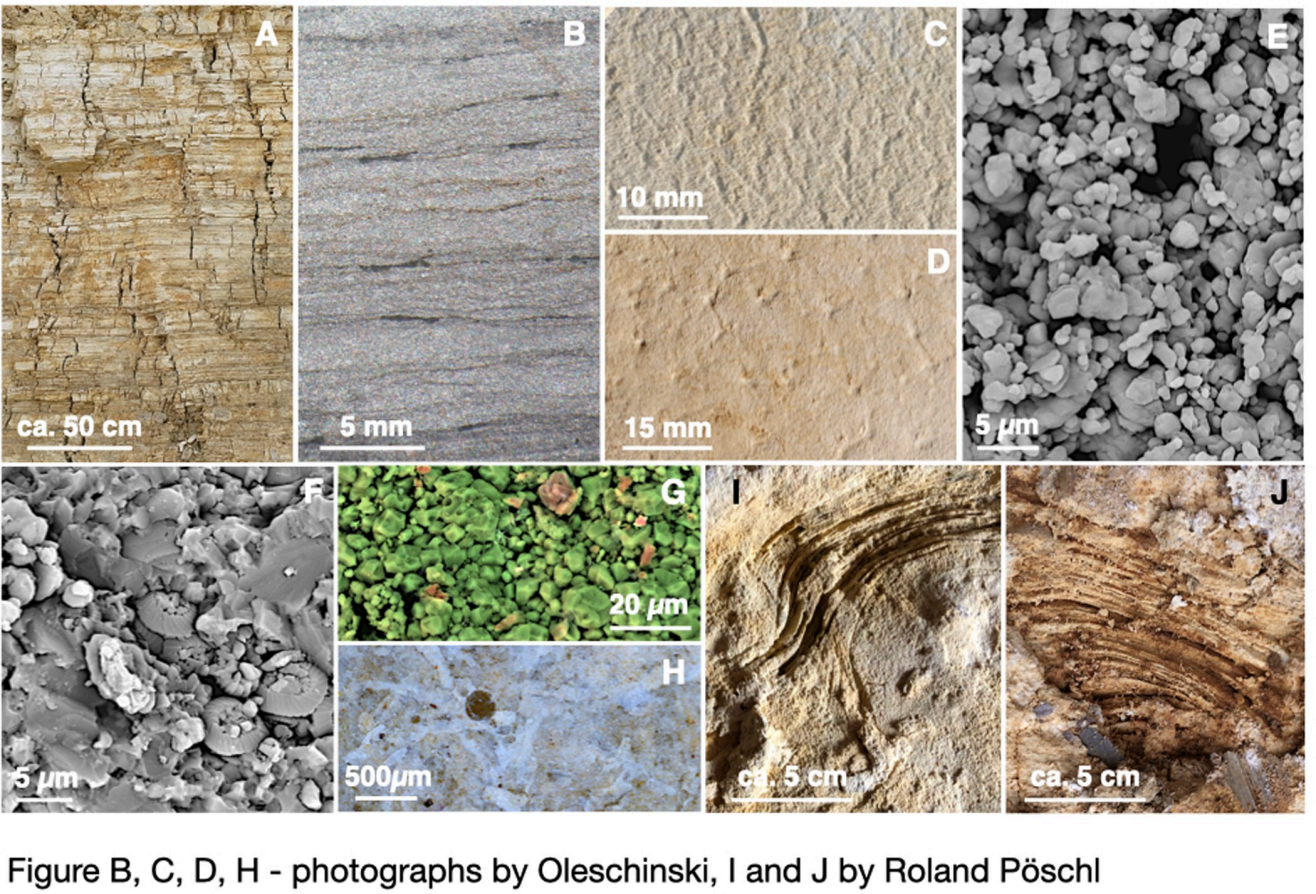
Images of plattenkalk sediments of the Solnhofen archipelago. (A)—plattenkalks of the Ettling quarry. (B)—photomicrograph of a polished thin slab of a dense plattenkalk layer in cross section from the Blumenberg quarry near Eichstätt, with thin opaque layers rich in (?) organic material. (C) and (D)—surfaces of plattenkalk layers with impressions from former bacterial mats. (E)—backscattered electron SEM image of plattenkalk in cross section from the Ettling quarry to illustrate the high porosity of some plattenkalks and the grain size distribution of calcite particles. (F)—a plattenkalk horizon from a road exposure near the Blumenberg quarry in Eichstätt, with coccolites concentrated on layer surfaces. (G)—energy-dispersive electron image of a micritic limestone from the Ettling quarry; calcite in green, siliclastic material in red. (H)—photomicrograph of a plattenkalk layer surface near Eichstätt, with whitish calcite pseudomorphs. (I) and (J)—(bio)-laminar structures from the Mülheim quarry interpreted as stromatolites (photo courtesy Roland Pöschl 2019).

On the macro-scale, the fossil-bearing plattenkalks consist of centimeter-thick even-bedded layers composed of ~ 95% micritic calcite, occasionally inter-layered with shaly calcareous marls. For details of the Solnhofen facies and the local nomenclature see [20, 21, 31]. Many calcite horizons are separated by thin dark laminae (Fig 4B) enriched in organic material near-opaque in thin slabs. These laminae may be interpreted as former bacterial or algal mats (Fig 4C and 4D). Within and among individual lagoons variations are noted with respect to strength and porosity of the plattenkalk layers. For example, in the Ettling lagoon (Fig 4A) plattenkalks are more friable and less cemented than plattenkalks around Solnhofen-Eichstätt. In some lagoons like Schamhaupten the plattenkalks can be silicified [32] reflecting the proximity of siliceous sponge reefs.

On the micro-scale the largest calcite particles are less than 5 μm in diameter (Fig 4E), and many grains are sub-micron. There has been much speculation about their origins. Some of the smaller particles may have been precipitates of cyanobacteria [33], some may be detrital material mobilised by storm events [20, 21] while others may be inorganic precipitates from CaCO_3_ oversaturated warm seawater (cf. refs [5, 34]). Microfossils like coccolites or foraminifera are rare (Fig 4F) but do occur on some bedding planes [28], possibly indicating periodic ingressions into the lagoons of open ocean water. The high porosity of the sediments is noteworthy (cf. [28]). It could reflect the fact that Cretaceous sediments are missing and that Tertiary sediments (if they were deposited at all) were thin, i.e. that post-Jurassic compaction by sediment overload was minor. Detrital siliciclastic material appears to be rare (Fig 4G).

**Fig 5:**
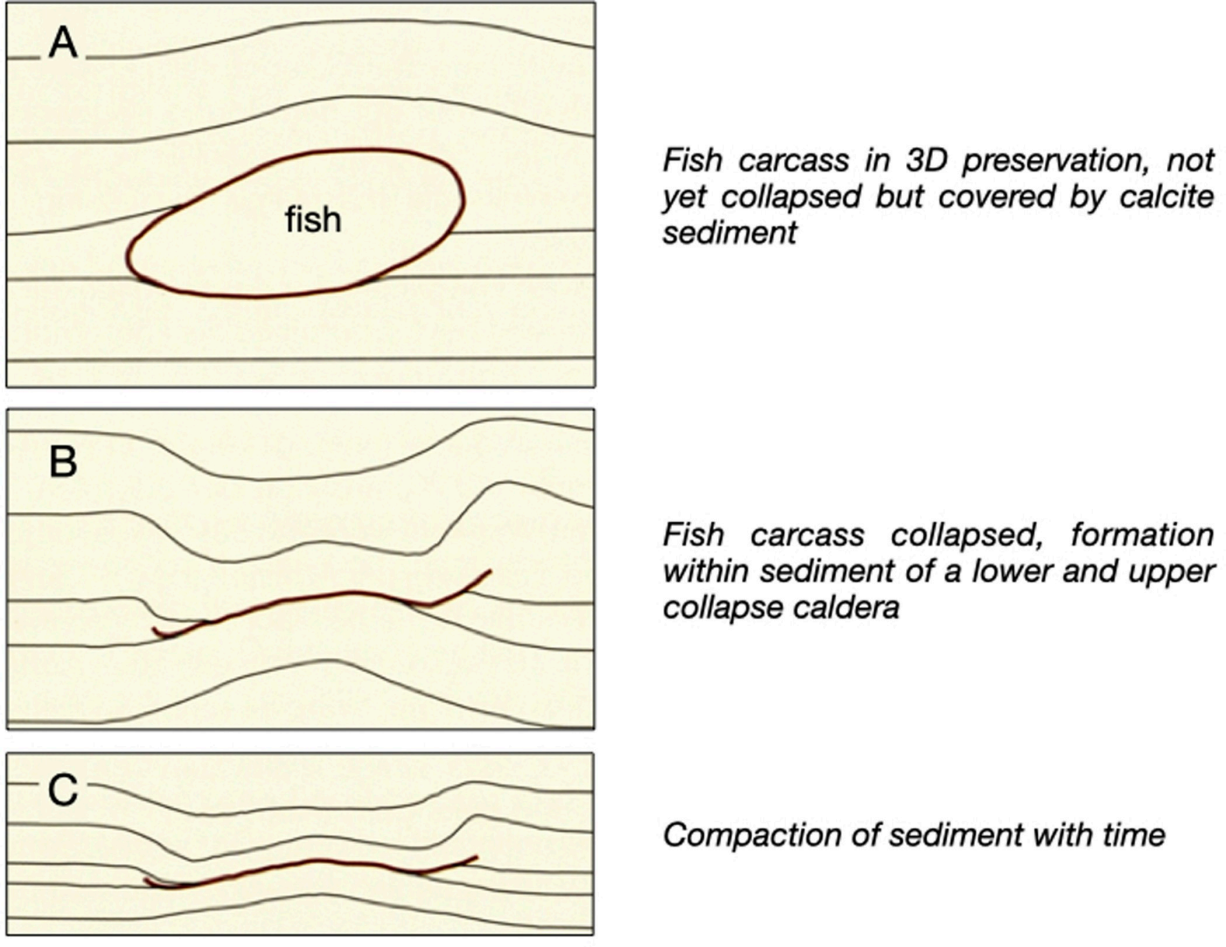
A cartoon modified from Viohl [21] to illustrate the compaction of a fish carcass covered by micritic calcite ooze. For implications on sedimentation rates see text.

There is no agreement on sedimentation rates and modes. Barthel [16] estimated that approximately 100 m of plattenkalk at Solnhofen were deposited within 250,000 years, corresponding to an average sedimentation rate of 0.4 mm per year. Park and Fürsich [35] argued that they identified in the Solnhofen plattenkalks Milankovich cycles and derived an average sedimentation rate of 0.048 to 0.139 mm calcite per year. Keupp et al. [20] and Viohl [21, 22], on the other hand, favoured episodic and rather rapid calcite accumulation from suspensions mobilised by summer storm events. The bacterial mats would then mark hiatuses, i.e. time periods of low sedimentation during the winter months. Viohl [21] underpins his sedimentation model with an informative cartoon that is reproduced in Fig 5, that documents the compaction rate of a fish carcass in 3D covered by calcite ooze. Obviously, the carcass collapsed only after several cm of sediment had been deposited above it. Gäb et al. [18] simulated a similar scenario experimentally at 8 bar hydrostatic pressure and found that a fish carcass under several cm of calcite sediment cover had largely collapsed after 77 days (cf. Fig 8 in ref. [18]). Their result applied to Fig 5 would rather argue for a fast, episodic sedimentation rate as envisioned by Viohl [21] since 3D preservation of fish fossils without disarticulation (cf. Fig 1) would be difficult to imagine at sedimentation rates of 0.1 to 0.5 mm per year.

Hardly any bioturbation is noted in the plattenkalks of Solnhofen [20]. Based on that observation, Fürsich et al. [36] argued that the bottom waters of most lagoons must have been anoxic. There is also speculation that the bottom waters of many Solnhofen lagoons were hypersaline although references to hypersalinity are largely circumstantial. Viohl [21] documented calcite pseudomorphs that he thought would outline former gypsum aggregates, implying salinities > 11.5 wt.% NaCl equiv. within CaSO_4_ * 2H_2_O saturation. In plattenkalks of the Mühlheim lagoon we noted laminated structures (Fig 4I and 4J) that resemble stromatolites, i.e. biosedimentary structures that in modern environments are assigned to hypersaline conditions [37, 38]. The dorsal curvature of many small fish (Fig 1C) is attributed by Seilacher et al. [11] and Pan et al. [39] to osmotic dehydration due to increased salinities and accumulation of putrefaction gases in body cavities. However, as long as no preserved syngenetic gypsum is identified, salinity stratification remains speculative.

## Methods

Sedimentation experiments were carried out to determine the settling rate of calcite from calcite suspensions. Calcite powders (35 g) with grain sizes of 2.2 to 4.4 μm were sedimented in volumetric flasks for 170 days from 100 ml solution. To address the possibility that many bottom waters in the Solnhofen lagoons were hypersaline, salinities were varied between zero and 14 wt.% NaCl. Prior to sedimentation the calcite suspensions were thoroughly homogenised, then sealed by plastic foils to avoid evaporation. From the volumes of the calcite oozes after 170 days and the zero-porosity volume of 35 g calcite (12.9 cm^3^) the porosity Φ is derived in percent, defined as ρ particles - ρ bulk suspension / (ρ particles - ρ pore solution) * 100. All solutions were halite-undersaturated. Hence, the only solid phase that was sedimented in the trials was calcite.

To investigate possible thixotropic behaviour of calcite oozes, the rheological properties of three calcite suspensions with zero, 3.2, and 9 wt.% NaCl were measured with an oscillation viscosimeter of the type MCR501 (Anton Paar) at the RTWH Aachen. The viscosimeter consists of a concentric cylindric sample vessel in which a cylindric metal spindle oscillates with a given angular frequency ω (in Hz) and amplitude γ ([40, 41], see also S1 Fig). The suspensions contained 40 vol.% micritic calcite and are located in the gap between the sample container and the oscillating spindle. The force necessary to move the spindle in the suspension is the shear stress τ. From the given deformation and the resulting shear stress a storage modulus G’ and a loss modulus G’’ are calculated, both given in units of Pascal. The storage modulus G’ describes the deformation energy that is stored in the sample during shearing, that can be recovered from the sample after relaxation. It thus quantifies the viscoelastic part of a suspension. The loss modulus G’’ is a measure for the energy lost during deformation in the form of heat. The ratio of both moduli describes the ability of a substance to store mechanical energy. For low-viscosity substances like water, G’’ > G’ and the storage modulus is zero since water cannot store mechanical energy. For substances with viscoelastic behaviour, G’ > G’’ and the moduli change with deformation amplitude and frequency depending on the viscoelastic properties of the substance.

Rheological properties were measured with an amplitude sweep profile that consisted of three measurement cycles with three successive shear amplitudes γ (in % where a 180° oscillation is defined as 100%). In the first, low shear phase (γ = 0.1%) a small shear deformation was applied to represent the resting phase of the suspension without significant mechanical stress. In the second, high-shear phase the shear amplitude γ was raised to 25% in order to test if and to what extent viscosities are reduced under high strain. After that deformation, the third, low-shear phase back to the initial γ = 0.1% enabled structural reorganisation of the suspension back to its original resting state (cf. Fig 2). The oscillation frequency ω was kept constant throughout at 1 Hz.

## Results

**Fig 6:**
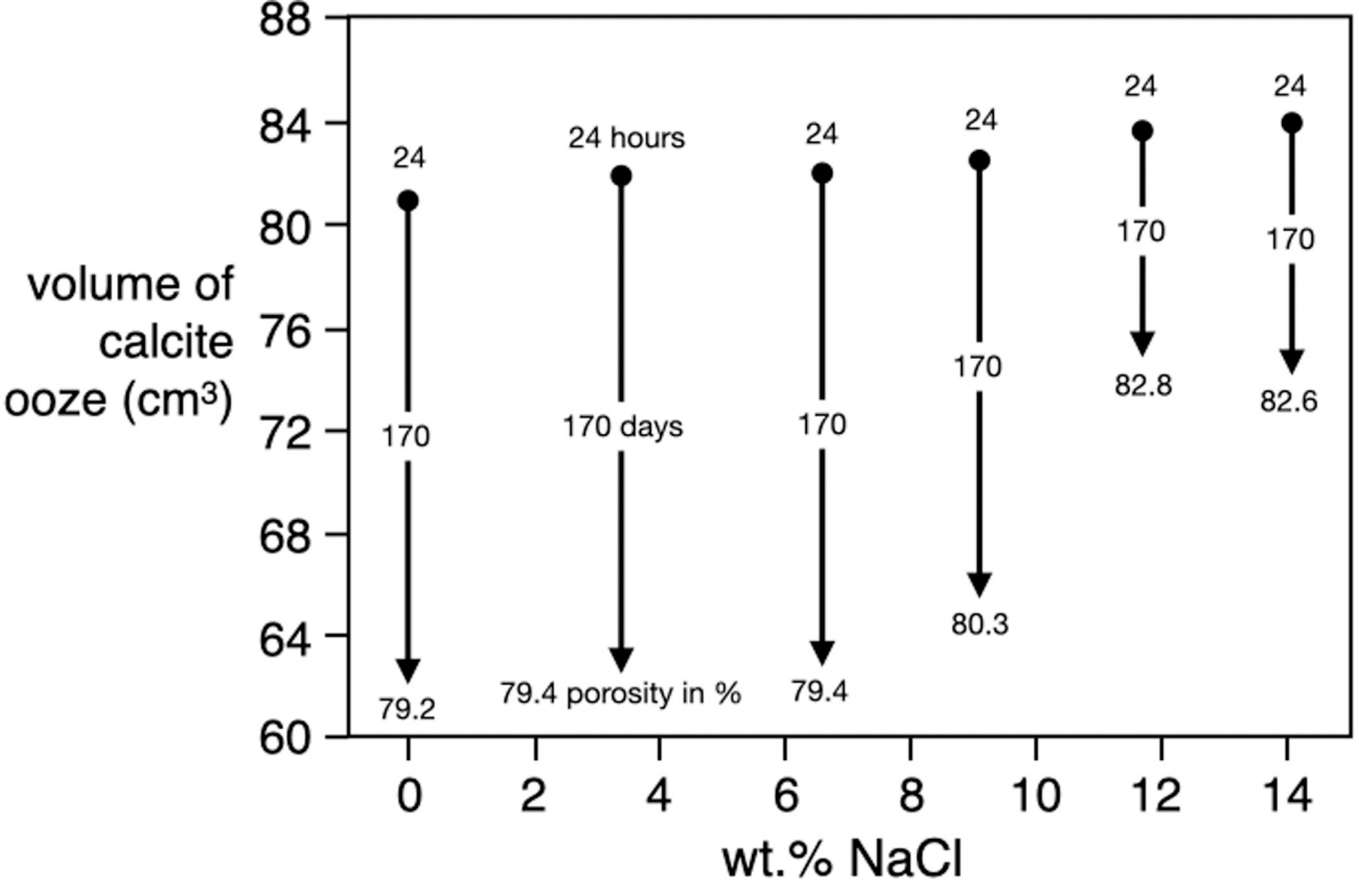
Compaction of micritic calcite suspensions with time as a function of salinity of the pore waters.

Sedimentation rates depend on the salinity of the solutions (Fig 6). After 170 days the suspensions with zero to 9 wt. % NaCl show similar degrees of compaction to around 80% porosity whereas the two suspensions with 11.8 and 14.2 wt. % NaCl were slightly less compacted with porosities around 82.7%. That may not appear a major difference, however, when we extrapolate runtimes to geologically relevant periodes of time, then calcite grains deposited from hypersaline conditions may end up forming more porous and more friable plattenkalks than calcite deposited from less saline solutions (see below). At the very beginning of a settling experiment, however, high salinities appear to promote settling rates. All solutions with NaCl were clear after 24 hours, while the zero NaCl suspension remained turbid for several days.

**Fig 7:**
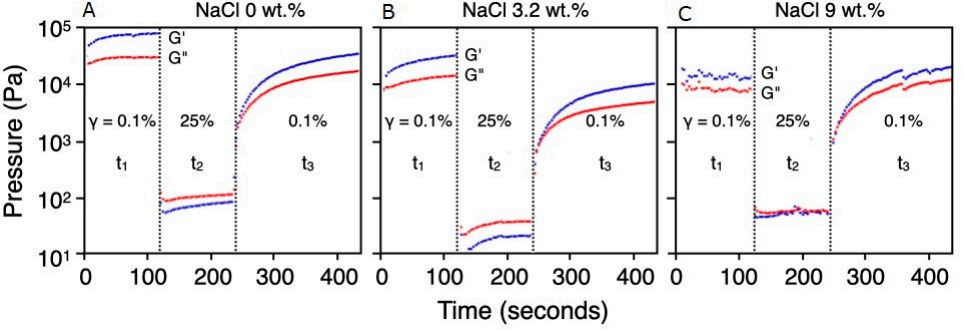
An amplitude sweep profile to measure the rheological properties of three calcite suspensions. Storage moduli G’ in blue, loss moduli G” in red. For details see text.

The results of the rheological measurements are shown in the form of amplitude sweep profiles in Fig 7. In the first cycle t_1_ with γ = 0.1%, the zero and 3.2 wt.% NaCl suspensions showed slight increases in G’ and G’’ (Fig 7A and 7B). Presumably, deformation in this low shear phase led to a reorganisation of structures (Fig 2) that had been disturbed when the suspensions were filled into the rheometer. If during this low shear phase G’ > G’’, which was the case for all trials, a suspension at rest behaves like a solid. In the 9 wt.% NaCl experiment (Fig 7C) fluctuations during t_1_ with respect to G’ and G’’ were found unexpectedly large.

After raising γ to 25% during t_2_, G’ and G’’ decreased in all suspensions by several orders of magnitude. At this high shear rate G’’ > G’, and in the 9.1 wt.% NaCl experiment both G’’ and G’ were found near-overlapping. During t_2_, all suspensions regardless of salinity behaved like low-viscosity liquids.

After returning at t_3_ to the original shear amplitude of γ = 0.1% the structures of all suspensions showed structural re-arrangements with G’ > G’’. With time, all suspensions increasingly behaved like viscoelastic solids. Due to time constraints, we have only been able to follow the structural recovery phase during t_3_ for about 500 sec and display in Fig 7 only the first 200 sec. However, extrapolation of G’ and G’’ beyond t_3_ suggests that with time all three suspensions would eventually have returned to their initial states but how quickly this would happen is not possible to quantify.

## Discussion

### Influence of salinity on compaction rates

Settling rates of calcite grains out of hydrous suspensions are influenced by grain sizes and salinities of the solutions. Minerals have surface charges, hence the smaller a grain the higher the contribution of surface energy to total energy. In the case of the calcite lattice, we assume that the negative charges are concentrated on crystal faces with a high CO_3_^2-^ occupation density, while positive charges are concentrated on crystal faces where Ca^2+^ prevails. In aqueous solutions, these surface charges are charged-balanced by OH^-^ and H^+^ ions depending on the pH [42]. In zero-salinity solutions this leads to the formation of rigid hydrate layers of chemically bound OH^-^ and H^+^ ions around calcite particles, and this hydrate layer could promote grain adhesion and rapid sedimentation rates when grain sizes are small. The higher the surface to volume ratios of the grains, the more significant surface effects will be.

With the addition of sodium chloride, additional ions are introduced, and these ions could neutralise even further remaining surface charges. In saline solutions, another, more diffuse but wider boundary layer may develop around calcite particles, and these layers are probably composed of hydrated Na^+^ and Cl^-^ ions [42]. It is possible that these additional hydrate shells statistically increased the spaces around calcite particles and inhibited compaction, as observed in Fig 6, and that for this reason we see after 170 days the lowest settling rates (highest porosities) in the highest salinity suspensions.

Settling rates could also be influenced by density differences between calcite particles and pore solution, since NaCl in solution increases the density of the pore water. For example, brines with 14 wt.% NaCl are about 10% denser than pure H_2_O. Relative to calcite, this results in a density difference with our highest salinity H_2_O-NaCl brine in Fig 6 compared to pure H_2_O, of 6.4%.

For each salinity, at least two trials were carried out to test reproducibility. With respect to porosity, we noted differences in the 2 to 4 relative percent between apparently identical experiments, which we attribute to grain size variations. When handling calcite powder, which was available in charges of 1 kg, it is inevitable that, with time and continued shaking and vibration, the material is sorted according to grain size [43, 44]. The smaller the average grain sizes, the stronger the effect in percentage terms. Although we aimed at homogenising the bulk calcite charges as thoroughly as possible before calcite powder was added to the solutions, we cannot ensure that the grain size distributions in all settling experiments were identical. Nonetheless, based on the results in Fig 6 we suggest that hypersaline water chemistries do decrease the compaction rate of calcite grains deposited from those brines, and eventually result in higher porosities of the resulting carbonate layers.

### Thixotropy

Thixotropic substances react with a reduction in viscosity when they are subjected to stress and return to their initial viscosities over time when the stress is relieved. If we apply this criterion to our rheological measurements, all suspensions studied are thixotropic. Unfortunately, thixotropy cannot be assigned an absolute value but is a relative parameter [41], but since we investigated three suspensions with differing salinities we may attempt to derive semi-quantitative parameters from the profiles in Fig 7 (cf. Table 1) to identify differences in rheological behaviour.

**Table 1:** Semi-quantitative parameters derived from the rheological results.

Parameter	Zero-NaCl	3,2 wt. % NaCl	9 wt. % NaCl
1. recovery ratio, % regeneration of G’ at 430 sec relative to t1	41	32	119
2. shear thinning index: G’ at t1 / G’ at t2	1024	1548	255
3. G’ max–G’ min end of t1, in Pa	81855	32485	13861
4. absolute pressure of G’ in Pa, end of t1	81930	32497	13909

Parameter 1 in Table 1 (recovery ratio) quantifies in percent how quickly a suspension returns to the initial state after deformation at t_1_. The recovery rate is listed by Mezger [41] as one of the parameters most indicative of thixotropic properties, as it takes into account the time dependence of structural recovery. For time constraints, we could not wait for complete structural recovery (i.e., G’ at t_3_ = G’ at t_1_), so we chose a reference time at 430 sec. Accordingly, the suspension with 9 wt.% NaCl would have to be assigned the highest thixotropy. However, the question is how useful a recovery of 119% (Table 1) is. The deformation profile of the 9 wt.% NaCl suspension during t_1_ was rather unsteady, indicating that this suspension was still far from its equilibrium resting state when the measuring spindle was inserted, and measurements commenced; that this suspension only approached its structural resting state during deformation at t_3_.

Parameter 2 in Table 1 quantifies the relationship between G’ during the low shear phase at t_1_ and G’ during the high shear phase at t_2_. That ratio is also known as the shear thinning index [45]. It quantifies the degree of shear thinning under stress. Accordingly, we would assign with parameter 2 the 3.2 wt.% NaCl experiment the strongest structural degradation. However, if instead of a quotient we calculate the difference between G’ at t_1_ and t_2_ (parameter 3 in Table 1)—perhaps justified because in absolute terms G’ at 3.2 wt.% NaCl is an order of magnitude lower than in the zero-salinity experiment—then the zero-NaCl suspension would be the one with the most pronounced thixotropy.

Parameter 4 in Table 1 quantifies the maximum achieved strength of a suspension at the end of t_1_, prior to increasing γ to 25%. Applying this parameter, the zero-salinity suspension would have the strongest structural strength and thus the most pronounced thixotropy. It should be noted though that all suspensions had only about 20 min time to regenerate structurally after they were filled into the rheometer. Perhaps this period was too short?

In summary, CaCO_3_ suspensions with particle sizes between 2.2 and 4.4 μm behave thixotropically, confirming observations by Freundlich [26]. All suspensions investigated showed a tendency to return to the initial viscoelastic state following exposure to high strain during t_2_. However, as mentioned above we cannot guarantee that the calcite oozes had reached maximum structural strength after they were filled into the rheometer and before stress was applied. One indication is that during the low-shear phase at t_1_, G’ and G’’ in all experiments increased with time. Perhaps the low strain rate during t_1_ promoted the regeneration of the structures (Fig 4) that had been disturbed prior to the measurements when the sample suspensions were added to the rheometer.

Regarding salinity effects, some ambiguity remains, and a clear influence of salinity on rheologic properties is less evident than desired. The observation that the 9 wt.% NaCl suspension had difficulty in regenerating structurally after deformation might indicate that high electrolyte concentrations in pore solutions do counteract a high structural strength. In the sedimentation experiments we also observed the lowest compaction rate of calcite in the highest salinity brines, and perhaps there is a correlation here. We therefore tend to assign the most pronounced thixotropy to the zero-salinity suspension and propose that elevated salinity slightly counteracts thixotropic behaviour. Accordingly, in these sediments we consider the grain size to be the main course for the thixotropic properties.

## Conclusions

We confirm early assumptions by Freundlich [26] that calcite suspensions with micritic grain sizes can exhibit thixotropic behaviour. Calcite oozes with distinctly thixotropic properties would solidify immediately after deposition. Even a few millimeters thick could effectively isolate a fish carcass from the overlying water column and create a local microenvironment favourable for preservation. We agree with Viohl [21, 22] and Keupp et al. [20] that the sedimentation of plattenkalk sediments in the Solnhofen lagoons was episodic. Optimal conditions for preservation and transfer of carcasses to the geological record are elevated salinities [18] and rapid, albeit episodic, sedimentation. Thixotropic behavior helps to promote diagenetic consolidation even if the sediment cover is still thin. Thus, we reiterate the initial assumptions that, in addition to the factors highlighted by Gäb et al. [18], excellent conservation of the fossils requires rapid coverage by sediment. Due to the slow settling rate shown in our experiments and the presence of bacterial mats on the layer surfaces, it can be assumed that the basins in the Solnhofen area were characterized by low average sedimentation rates. Thus, deposition of the thixotropic layers must therefore have taken place in short periods of time in the course of a rapid sedimentation, as it can be derived from the sedimentary observations, such as shown in Fig 5. This assumption corresponds to the theory of Keupp et al. [20] and Viohl [21, 22], according to which summer storm events lead to the mobilization of calcite and therefore to a high and rapid sedimentation.

In addition, thixotropic behaviour may also have influenced the facies of the plattenkalks in the Solnhofen lagoons. The greater the tendency toward thixotropic behaviour, the faster diagenetic hardening should have proceeded after the calcite suspensions were deposited. If thixotropic properties of a micritic calcite suspension correlate with the salinity of the pore waters, as suggested by the deformation spectra in Fig 7 and the semi-quantification in Table 1, we might also explain facies variations among the plattenkalks of the Solnhofen lagoons with salinity variations. The solid and dense plattenkalks found around Eichstätt and Solnhofen would then have precipitated out of micritic suspensions with moderate salinities. The more porous, more crumbly, and more friable plattenkalks at e.g. the Ettling locality could have been sedimented from distinctly hypersaline bottom waters. Perhaps it is not a coincidence that fossils in the Ettling sediments are quite rare but unusually well preserved, in part with original pigmentation [5]. That observation may support unusually saline water compositions in the Ettling lagoon. Gäb et al. [18] showed that salinities > 3 times seawater are a good recipe for fossilisation. All this with some reservations, primary (syngenetic) gypsum needs to be identified in the plattenkalks of the Solnhofen archipelago before we can confirm that waters in the Solnhofen lagoons were indeed hypersaline and stratified with respect to salinity.

## Supporting information

S1 Fig Schematic representation of a concentric cylinder measuring system. (TIF)

S1 Table Table of the rheological results. (XLSX)
