# Supplementary figures and images for: Rheological properties of calcite oozes: Implications for the fossilisation in the plattenkalks of the Solnhofen-Eichstätt lagoons in the Franconian Alb, Germany
Source: PLoS One. 2021 Jun 2;16(6):e0252469. doi: 10.1371/journal.pone.0252469 (PMC8171948; doi:10.1371/journal.pone.0252469)

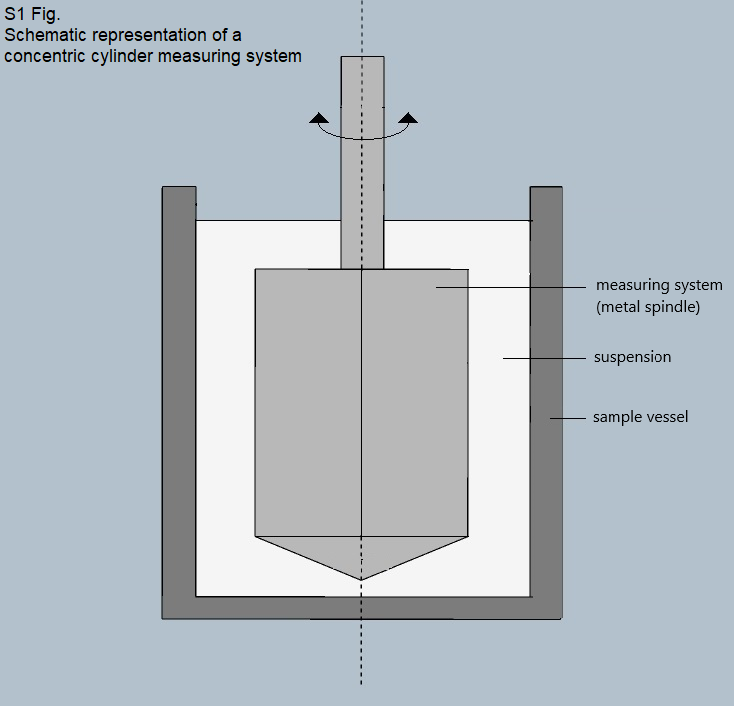

Supplement: S1 Fig — (TIF) [file pone.0252469.s001.tif]
